# Cancer/Testis Antigens as Biomarker and Target for the Diagnosis, Prognosis, and Therapy of Lung Cancer

**DOI:** 10.3389/fonc.2022.864159

**Published:** 2022-04-27

**Authors:** Ping Yang, Yingnan Qiao, Mei Meng, Quansheng Zhou

**Affiliations:** ^1^ Department of Pathophysiology, School of Medicine, Nantong University, Nantong, China; ^2^ Cyrus Tang Hematology Center, Jiangsu Institute of Hematology, Soochow University, Suzhou, China; ^3^ State Key Laboratory of Radiation Medicine and Protection, School of Radiation Medicine and Protection, Soochow University, Suzhou, China; ^4^ 2011 Collaborative Innovation Center of Hematology, Soochow University, Suzhou, China; ^5^ National Clinical Research Center for Hematologic Diseases, The Affiliated Hospital of Soochow University, Suzhou, China

**Keywords:** lung cancer, cancer/testis antigens, immunotherapy, car-t, cancer diagnosis, prognostic prediction, novel therapeutics

## Abstract

Lung cancer is the leading type of malignant tumour among cancer-caused death worldwide, and the 5-year survival rate of lung cancer patients is only 18%. Various oncogenes are abnormally overexpressed in lung cancer, including cancer/testis antigens (CTAs), which are restrictively expressed in the male testis but are hardly expressed in other normal tissues, if at all. CTAs are aberrantly overexpressed in various types of cancer, with more than 60 CTAs abnormally overexpressed in lung cancer. Overexpression of oncogenic CTAs drives the initiation, metastasis and progression of lung cancer, and is closely associated with poor prognosis in cancer patients. Several CTAs, such as XAGE, SPAG9 and AKAP4, have been considered as biomarkers for the diagnosis and prognostic prediction of lung cancer. More interestingly, due to the high immunogenicity and specificity of CTAs in cancer, several CTAs, including CT45, BCAP31 and ACTL8, have been targeted for developing novel therapeutics against cancer. CTA-based vaccines, chimeric antigen receptor-modified T cells (CAR-T) and small molecules have been used in lung cancer treatment in pre-clinical and early clinical trials, with encouraging results being obtained. However, there are still many hurdles to be overcome before these therapeutics can be routinely used in clinical lung cancer therapy. This review summarises the recent rapid progress in oncogenic CTAs, focusing on CTAs as biomarkers for lung cancer diagnosis and prognostic prediction, and as targets for novel anti-cancer drug discovery and lung cancer therapy. We also identify challenges and opportunities in CTA-based cancer diagnosis and treatment. Finally, we provide perspectives on the mechanisms of oncogenic CTAs in lung cancer development, and we also suggest CTAs as a new platform for lung cancer diagnosis, prognostic prediction, and novel anti-cancer drug discovery.

## Introduction

Lung cancer is the leading type of malignant tumour associated with cancer-caused death worldwide. In 2020, lung cancer occurred in 2.2 million people and resulted in 1.8 million deaths globally. It is the primary cancer-related cause of death in men and is the secondary cause in women (1). Although great efforts to fight lung cancer have been made in recent decades, the 5-year survival rate for lung cancer patients is only 18% (2). The main reasons for the poor outcome of lung cancer are the lack of a highly specific and sensitive biomarker for early diagnosis of the disease and the absence of effective drugs for lung cancer therapy (3–5).

It is well established that lung cancer patients harbour many driver gene mutations, such as K-ras (6, 7), EGFR (8), EML4-ALK (9), and CLIP1-LTK (10), which contribute to the initiation and progression of lung cancer. Accordingly, several oncogenic genes have been used as biomarkers for lung cancer diagnosis; however, the sensitivities of K-ras-G12C, EGFR, EML4-ALK, and CLIP1-LTK in lung cancer patients are only 14%, 46%, 4%, and 0.5%, respectively (6–10). K-ras-G12C and EGFR-targeted therapeutics against lung cancer appear promising in patients with these mutants and have prolonged survival time in the short term (6, 8, 10). However, the 5-year overall survival rate of lung cancer patients has only improved from 16% 10 years ago to 18% now, implying that merely targeting these mutant genes is not enough to conquer lung cancer (1–5, 11, 12). Thus, sensible biomarkers and effective drugs against lung cancer are highly desirable.

Emerging evidence shows that oncogenic gene amplification plays a critical role in cancer progression and that numerous oncogenic genes are aberrantly overexpressed in various types of malignant tumours, including lung cancer. More importantly, the gene dosage has recently been recognized as the most important factor that governs tumour initiation, metastasis and progression (13–18). Therefore, in addition to gene mutation, overexpressed oncogenic genes should be selected as new biomarkers and targets in cancer diagnosis and therapy. Numerous oncogenic genes are abnormally overexpressed in lung cancer, including Myc, TERT, NSD3 and various cancer testis antigens (CTAs) (14–17, 19, 20).

The CTAs are named according to their expression pattern, which is restricted in the testis after embryonic development but is not expressed in other normal adult tissues. So far, more than 700 CTAs have been identified to have aberrant overexpression in various cancers, and more than 90 CTAs have been detected in lung cancer cells or tissues. Collectively, these 90 CTAs are aberrantly expressed in more than 95% of lung tumour tissue samples from lung cancer patients, while most of them are silenced in normal lung tissue samples (14–17, 19, 20).

Oncogenic CTAs play an important role in lung cancer initiation and progression by enhancing numerous oncogenic gene expressions and activating multiple signalling pathways, consequently promoting lung cancer cell proliferation, migration, invasion, metastasis and epithelial-mesenchymal transformation (EMT) (20). Because of the high antigenicity and immunogenicity of CTAs, several of them, such as the MAGE-A family, BCAP31, and the LY6 family, have been considered as biomarkers for lung cancer diagnosis and prognostic prediction (21–23). Additionally, several oncogenic CTAs, including SP17, BORIS, and CAGE, have been targeted for novel anti-lung cancer drug discovery and have provided encouraging results in *in vitro* and *in vivo* studies (24–27). CTA-based vaccines, chimeric antigen receptor-modified T cells (CAR-T), and small molecules have been used in lung cancer treatment in pre-clinical and early clinical trials and have exhibited encouraging outcomes (20). However, there are still many hurdles to be overcome before these therapeutics can be routinely used in clinical lung cancer therapy.

In this review, we summarize the rapid progress in research on oncogenic CTAs in lung cancer initiation and progression with a focus on CTAs as biomarkers for lung cancer diagnosis and prognostic prediction and as targets for novel therapeutics. We also address the current challenges and opportunities in CTA-based lung cancer diagnosis and treatment. Finally, we offer perspectives on CTA-based lung cancer diagnosis and prognostic prediction, and CTAs as a novel platform for developing therapeutics.

## The Role of Cancer Testis Antigens in Tumorigenesis and Metastasis of Lung Cancer

### Oncogenic CTAs Are Aberrantly Overexpressed in Lung Cancer

**Figure 1:**
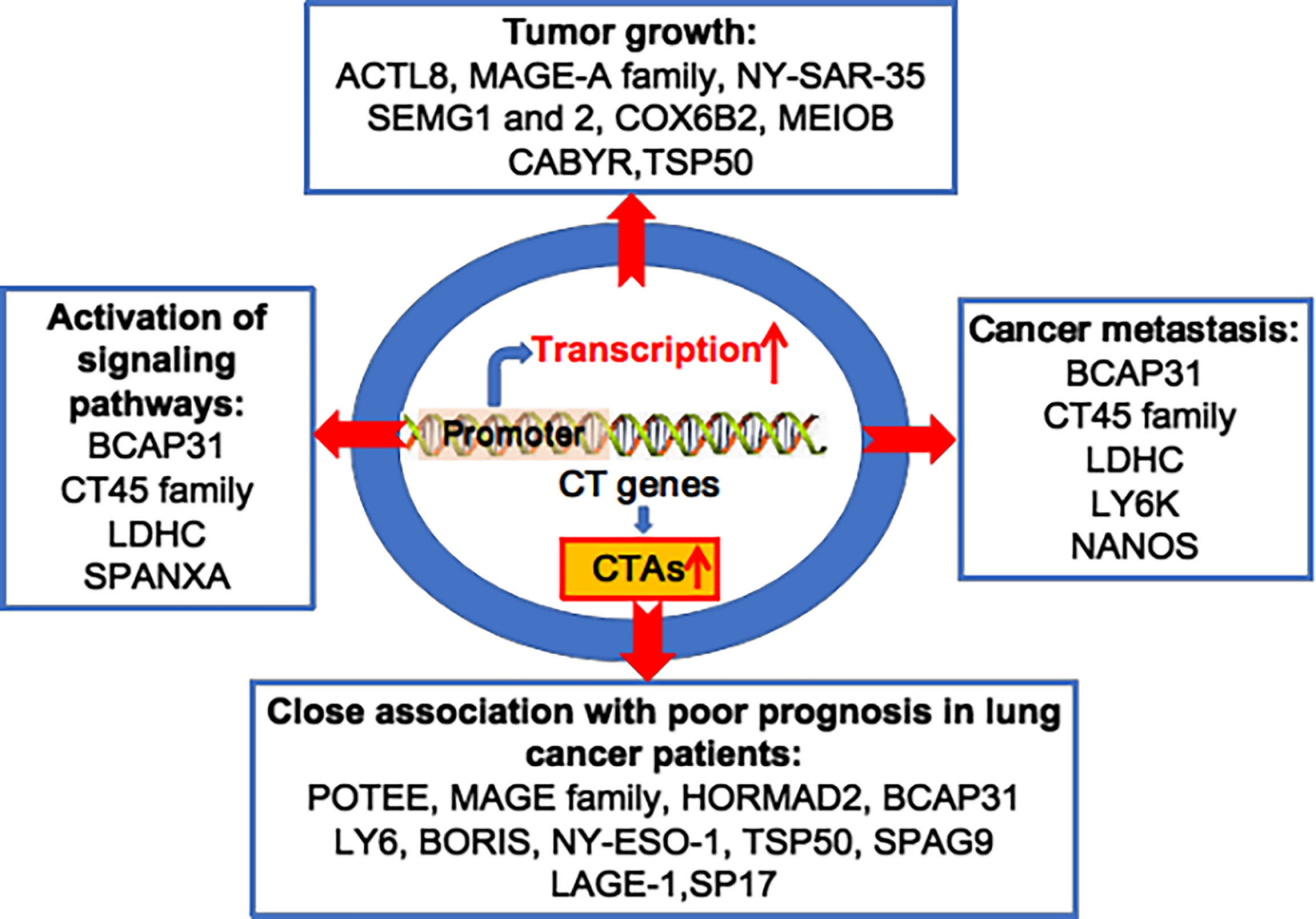
Oncogenic CTAs drive lung cancer initiation and progression. Various CT genes are aberrantly activated, and overexpression of CTAs plays important roles in lung tumour growth, cancer metastasis, activation of multiple signalling pathways, and is closely associated to poor prognosis in lung cancer patients.

Since Holden et al. first reported on CTAs in 1977, more than 700 CTAs have been identified, with over 90 being abnormally overexpressed in lung cancer (20, 28–31). In 2005, Chen et al. found that the CT45 family and other CTAs were overexpressed in lung cancer cell lines and tissues (32). Chen et al. reported that several CTAs, including cancer/testis antigen 1B (NY-ESO-1), MAGE family member A4 (MAGEA4) and sarcoma antigen 1 (SAGE1), were overexpressed in 1023 non-small-cell lung cancer (NSCLC) cases (33). Dijana et al. reported that 96 CTAs, such as TKTL1, TGIF2LX, VCX, and CXORF67, were highly expressed in 199 NSCLC tumour tissues compared to 142 normal lung tissue samples (34). Additionally, oncogenic MAGE-A6 was overexpressed in lung cancer patients with resistance to the drug vandetanib, compared to those susceptible to vandetanib (35). Furthermore, tumorigenic NANOS3 is overexpressed in 94.7% of tumour tissues from 94 NSCLC patients (36). It has been reported that many other CTAs are abnormally overexpressed in lung cancer, including ankyrin domain family member E (POTEE), Sperm-associated antigen 9 B (SPAG9), cell receptor associated protein 31 (BCAP31), CCCTC-binding factor like (BORIS), bromodomain testis associated (BRDT), calcium binding tyrosine phosphorylation regulated (CABYR), lymphocyte antigen 6 family member K (LY6K), cancer/testis antigen 2 (LAGE-1), cancer/testis antigen family 45 (CT45), cancer/testis antigen family 47 (CT47), DDB1 and CUL4 associated factor 12 (TCC52), FMR1 neighbour (NY-SAR-35), and the X antigen family member (XAGE) and SSX families (37) (**Figure 1**).

### Oncogenic CTAs Promote Lung Cancer Cell Tumorigenesis

Several oncogenic CTAs contribute to tumorigenesis of malignant tumours, including lung cancer. The MAGE-A family contains 12 members named MAGE-A1 to A12 and is one of the most frequently overexpressed CTA families in malignant tumours. MAGE-A family is abnormally overexpressed in 69.2% of patients suffering from lung squamous carcinoma, with high immunogenicity and tumour specificity (38). Thus, MAGE-A is an ideal biomarker for lung cancer diagnosis and a target for immunotherapy. Generally speaking, the overexpression of MAGE-A family members promotes tumorigenesis. It has been reported that MAGE-A inhibits apoptosis and promotes proliferation in multiple myeloma through the regulation of BIM and p21Cip1 (39). Overexpression of MAGE-A induces EMT by upregulating key genes, such as β-catenin (40). MAGE-A is involved in the protection of the male germline against environmental stress to ensure reproductive success under non-optimal conditions. Unfortunately, this function of MAGE-As is hijacked by cancer cells, resulting in tumour cell survival under environmental stress (41). Several MAGE-A family members exert divergent roles in carcinogenesis. MAGE-A11 inhibits prolyl hydroxylase 2 (PHD2) enzymes and reduces the levels of hypoxia-inducible factors (HIFs), well-known transcription factors that promote the overexpression of angiogenic genes, thus inhibiting tumour angiogenesis. It has also been reported that MAGE-A11 gene knockdown decreases the expression of HIF-1 and downstream target genes (42–44). Hence, the enhancement of the function of MAGE-A11 in the regulation of HIFs needs to be verified.

MAGE-A4 induces lung cancer cell apoptosis, suggesting that it has a tumour-suppressive function (45). MAGE-A3 stimulates E3 ubiquitin ligase tripartite motif-containing protein 28 (TRIM28), resulting in down-regulation of p53 and cellular energy sensor AMP-activated protein kinase (AMPK) (46).

Actin like 8 (ACTL8) is highly expressed not only in lung cancer but also in various other types of tumours (47). ACTL8-knockdown inhibits lung cancer cell proliferation, colony formation, cell cycle progression, migration and invasion, and lung cancer cell A549 tumour growth *in vivo*, indicating that it exerts an oncogenic role in lung cancer cell tumorigenesis and progression (47). Overexpression of NY-SAR-35 promotes lung cancer cell proliferation and increases cell viability, migration and invasion (48). Semenogelins 1 and 2 (SEMG1 and 2) interact with the glycolytic enzymes-pyruvate kinase M2 (PKM2) and lactate dehydrogenase A (LDHA), increase the expression levels of these two partners, and elevate the protein levels of the membrane mitochondrial potential, resulting in increases in glycolysis, respiration and ROS production in several cancer cell lines, implying that SEMG1 and 2 enhance cancer cell energy metabolism and exemplify tumour cell oncogenic features (49).

Cytochrome c oxidase subunit 6B2 (COX6B2) is highly expressed in human lung cancer and is correlated with a reduced survival time in cancer patients (50). Overexpression of COX6B2 enhances the activity of cytochrome c oxidase complex IV and increases oxidative phosphorylation (OXPHOS) and NAD+ generation, leading to an increase in ATP production and robust tumour cell proliferation. Convincible, knockdown of COX6B2 elicits cell apoptosis or senescence due to a decrease in OXPHOS and mitochondrial membrane collapse in tumour xenograft mice (51).

Meiosis-related EECTG (MEIOB) plays a critical role in the development of cancer. Overexpression of MEIOB in lung cancer cells increases cell viability and malignant phenotypes; whereas the deletion of MEIOB inhibits lung cancer cell colony formation, growth, invasion and migration. The calcium-binding tyrosine phosphorylation-regulated gene (CABYR) is associated with anaerobic glycolysis-driven energy generation (52). Knockdown of CABYR-a/b significantly inhibits proliferation of lung cancer cells, attenuates Akt phosphorylation, decreases phosphorylated GSK levels, and increases the levels of p53 and p27 proteins (53). However, it has been reported that CABYR-a/b increases tumour necrosis factor receptor superfamily, member 10b (DR5) expression and sensitizes lung cancer cells to the tumour necrosis factor-related apoptosis-inducing Ligand (TRAIL)-induced apoptosis *in vitro* and *in vivo* (54).

Testes-specific protease 50 (TSP50) is specifically expressed in the testis and is also overexpressed in lung cancer tissues. Overexpression of TSP50 promotes cancer cell proliferation, colony formation and migration *in vitro*, while silencing of TSP50 inhibits tumorigenic capability, induces G0/G1-phase arrest, downregulates the expression levels of the cell cycle-relative markers CDK4, CDK6 and Cyclin D1, and upregulates the expression of the tumour suppressors p21 and p53 in lung cancer 95-D cells (55).

Several other CTAs, including BAGE, PD-L1, MAGE-3 and AKAP4, are significantly dysregulated in smokers and NSCLC patients compared to healthy people (56). Tumours from smokers or males have significantly higher transcript levels of lactate dehydrogenase C (LDHC) than non-smokers or females, implying that there is a vital role of LDHC in carcinogenesis (57).

### Oncogenic CTAs Enhance Lung Cancer Metastasis and Progression

Increasing evidence shows that oncogenic CTAs promote lung cancer metastasis and progression. For example, B cell receptor associated protein 31 (BCAP31) significantly increases lung cancer cell migration and invasion. Signalling pathway analysis shows that the Akt/m-TOR/p70S6K pathway is significantly activated by BCAP31. In addition, BCAP31 plays a role in lung cancer metastasis (21).

The CT45 family consists of nine members and is especially overexpressed in lung cancer tissues and not in normal lung tissues, suggesting that CT45 is a specific marker of lung cancer (58). CT45A1 is a proto-oncogene that drives overexpression of various oncogenic genes, such as c-kit, ALDH1, CXCR4 and Twist1. Overexpression of CT45A1 promotes breast cancer metastasis to the lung (59). Convincingly, CT45A1 silencing suppresses the proliferation, metastasis and invasion of lung cancer cells by diminishing the ERK/CREB signalling pathway (60). CT45A2 also promotes cell proliferation and motility *via* the transcriptional factor TCF4 and β-catenin signalling pathways (61).

LDHC is a specific isoenzyme of the LDH family and is overexpressed in lung cancer. The transcription factor Sp1 and CREB-mediated overexpression of LDHC promote lung cancer cell proliferation *in vitro* and metastasis *in vivo* (62, 63). In addition, LDHC induces lactate and ATP production and upregulates c-Myc, cyclin D1, matrix metalloproteinase (MMP)-2, MMP-9, Vimentin, Twist, Slug and Snail. It activates the PI3K/Akt/GSK-3β-signalling pathway and promotes lung cancer cell proliferation and EMT (63).

The NANOS family contains two members named NANOS1 and NANOS3. NANOS1 is involved in the acquisition of the invasive properties of lung tumour cells *via* promoting the overexpression of matrix metalloproteinase (MMP)-14 (64, 65). NANOS3 is also overexpressed in invasive lung cancer cells. The overexpression of NANOS3 enhances lung cancer cell EMT. Moreover, NANOS3 represses E-cadherin at the transcriptional level, upregulates vimentin post-transcriptionally and protects the mRNA of vimentin from degradation (36) (**Table 1**).

**Table 1:** Oncogenic CTAs promote lung cancer initiation and progression.

CTA name	Function	Ref
NANOS3	Promote lung cancer invasion by inducing EMT	(36)
AKAP4	Promote lung cancer lymphatic metastasis	(66)
MAGE-A family	Oncogene, boost lung cancer initiation and progression	(67)
CT45	Proto-oncogene, promote lung cancer metastasis	(60, 61)
LDHC	Induce lung cancer cell proliferation and metastasis	(63)
ACTL8	Enhance cancer cell migration, invasion, and stemness	(47)
BCAP31	Promote lung cancer cell metastasis	(21)
SEMG1 and 2	Induce oxidative stress and tumorigenic metabolism	(49)
NY-SAR-35	Promote cancer cell proliferation, migration, and invasion	(48)
COX6B2	Enhance cancer cell proliferation and survival	(51)
MEIOB	Oncogene, promote cell malignant transformation	(68)
CABYR-a/b	Promote cell proliferation and decrease chemo-sensitivity	(53, 54)
LY6K	Promote tumor cell proliferation and invasion	(69)
TSP50	Enhance cancer cell proliferation and invasion	(55)
BRDT	Promote lung cancer initiation	(70)

### Emerging Role of CTAs in Tumour Suppression

CTAs have divergent functions in the regulation of tumorigenesis. Apart from the oncogenic CTAs mentioned above, several other CTAs have recently been reported as tumour suppressors that can inhibit cancer cell proliferation, angiogenesis and metastasis (71). For example, testis-specific gene antigen 10 (TSGA10) suppresses cancer development in various types of malignant tumours by inhibiting HIF-1 expression, tumour cell metastatic capability, and metabolic activity in breast cancer (72). TSGA10 also diminishes the angiogenesis of human vascular endothelial cells. TSGA10 expression is significantly reduced in cancer patients and the downregulation of TSGA10 is associated with high VEGF levels, tumour angiogenesis and cancer metastasis (73–75). MiR-10b-3p and miR-23a reduce the expression of TSGA10, thus promoting cancer progression (76, 77).

In another instance, G-protein signalling 22 (RGS22) functions as a tumour suppressive CTA to exhibit tumour progression by inhibiting tumour cell invasion and metastasis in liver cancer, pancreatic adenocarcinoma and epithelial cancers (78, 79). MAGE-A4 exerts pro-apoptotic activity by binding to RING E3 ligases, p21Cip1, Miz1 and P53, resulting in DNA damage and tumour suppression (80, 81). The expression levels of sperm protein associated with the nucleus on the X-chromosome family members A (SPANXA) are much higher in low-invasive lung cancer cells than in high-invasive cells. Overexpression of SPANXA inhibits tumour cell invasion and metastasis *in vitro* and *in vivo* by suppressing the c-JUN-SNAI2 axis and upregulating E-cadherin (82).

Together, this evidence indicates an emerging role of CTAs for tumour suppression. Tumour-suppressive CTA research is still at an early stage. The mechanisms underlying CTA-mediated tumorigenesis and cancer metastasis remain to be elucidated. On the other hand, further investigation of tumour-suppressive CTAs will provide a new platform for developing novel therapeutics against cancer.

## CTAs as Biomarkers for Lung Cancer Diagnosis and Prognostic Prediction

### CTAs as Biomarkers for Lung Cancer Diagnosis

CTAs have high cancer specificity and sensitivity and researchers have explored using them as biomarkers for lung cancer diagnosis. For example, Shan et al. used a microarray consisting of 72 CTAs and six non-CTAs to screen for autoantibody biomarkers in NSCLC. A CTA panel of NY-ESO-1, XAGE-1, ADAM29 and MAGE-C1 had specificity and sensitivity values of 89% and 36%, respectively, in lung cancer patients (83). XAGE-1a and XAGE-1d belong to the XAGE-1 family and are overexpressed in lung cancer but are scarcely expressed in normal lung tissues, if at all. The levels of XAGE-1a and XAGE-1d in lung cancer patients are 1620 ng/L and 2510 ng/L, respectively, which are much higher than those of the widely used cancer marker CEA, suggesting that they are excellent biomarkers for the diagnosis of lung cancer (84).

Sperm associated antigen 9 (SPAG9) is also overexpressed in lung cancer. Additionally, the levels of SPAG9 autoantibodies in the serum of lung cancer patients are higher than in healthy controls (85). Thus, both SPAG9 and SPAG9 autoantibodies can be used in lung cancer diagnosis. AKAP4 expression increases significantly with the advancement of the tumour stage and is independent of age, gender, smoking history or cancer subtype, suggesting that AKAP4 is a highly accurate biomarker for the detection of early-stage lung cancer and prediction of lung cancer progression (66).

### Oncogenic CTAs as Biomarkers for Lung Cancer Prognostic Prediction

Oncogenic CTAs promote tumorigenesis and cancer metastasis; accordingly, researchers have studied whether CTAs are useful biomarkers for lung cancer prognostic prediction. For instance, the MAGE-A family is closely associated with poor prognosis in many types of cancer, including lung cancer (22, 86–88). Lung cancer patients with MAGE-A expression exhibit worse survival than those with MAGE-A-negative cases. The 5-year survival rates of lung cancer patients with positive or negative MAGE-A expressions are 59.4% and 78.7%, respectively, indicating that overexpression of MAGE-A results in an unfavourable prognosis (22, 86–88). Additionally, patients with MAGE overexpression have a worse prognosis than those with no MAGE expression (89).

**Figure 2:**
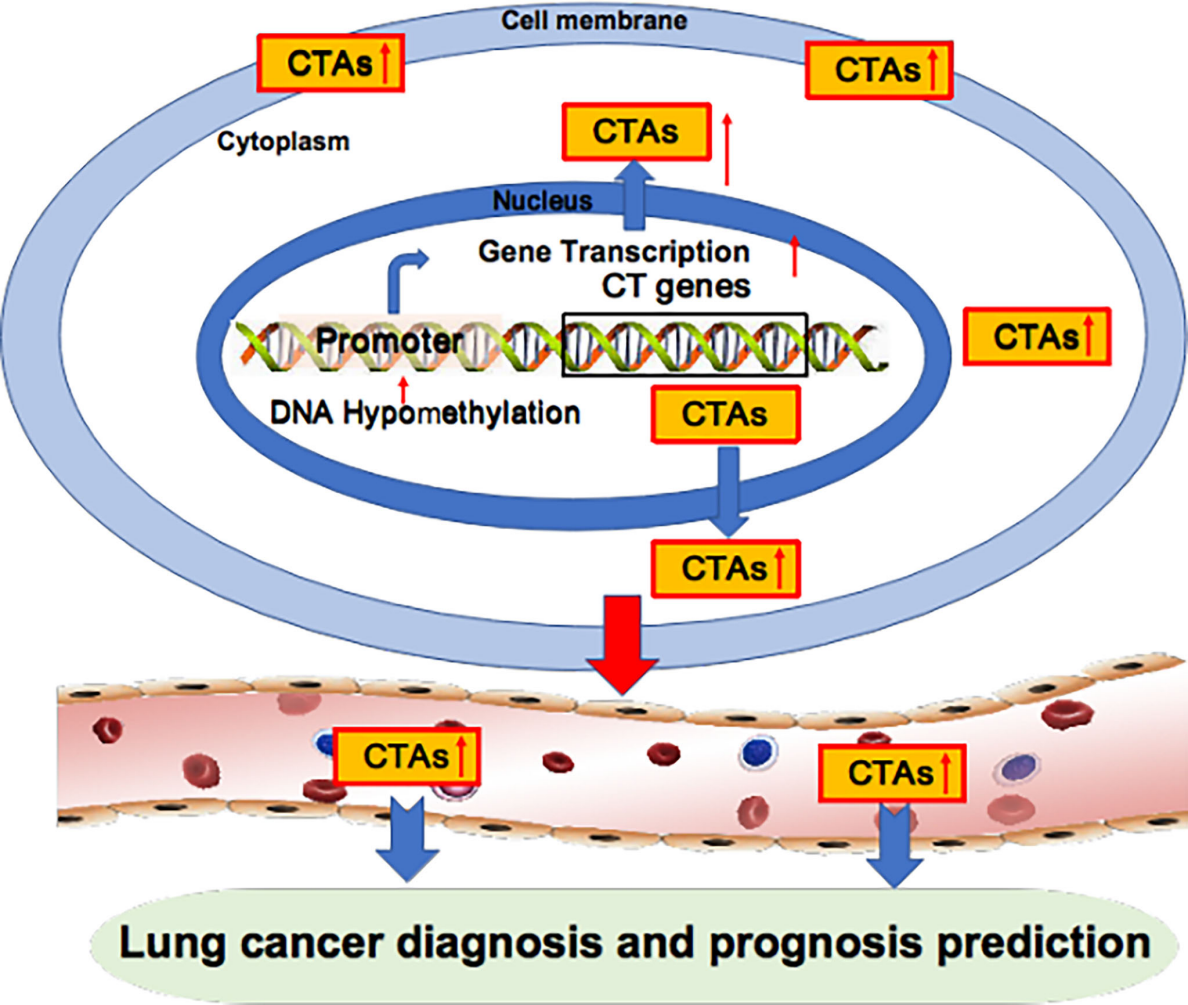
Oncogenic CTAs as targets for lung cancer diagnosis and prognosis prediction. Oncogenic CTAs are overexpressed due to hypomethylation of the gene promoter, resulting in robust gene transcription. CTAs are secreted from cancer cells and enter blood circulation. Thus, CTAs can be detected in the blood and used as biomarkers for lung cancer diagnosis and prognosis prediction.

Aberrant overexpression of BCAP31 is associated with histological grade, low overall survival and poor prognosis in lung cancer patients (21). The LY6 family, consisting of Ly6D, Ly6E, Ly6K and Ly6H, is also associated with poor survival in several cancer types, including lung cancer (23). Brother of the regulator of the imprinted site variant subfamily 6 (BORIS-sf6) is a negative prognostic predictor of lung cancer patients (24). Notably, the median survival times of patients with BORIS-sf6 overexpression or low expression were 26 weeks and 209 weeks, respectively; thus, BORIS-sf6 acts as a powerful prognostic predictor for lung cancer. TSP50 expression levels are higher in the advanced TNM stage in lung cancer patients (90). Kaplan−Meier analysis shows that disease-free survival and overall survival were significantly lower in lung patients with overexpressed TSP50 compared with those with low TSP50 expression, implying that TSP50 overexpression is associated with poor prognosis in lung cancer patients. Collectively, the overexpression of oncogenic CTAs in cancer is closely associated with poor prognosis in lung cancer patients (**Figure 2**, **Table 2**).

**Table 2:** Oncogenic CTAs are associated with poor prognosis in lung cancer patients.

CTA name	Expression rate in lung cancer	Related to poor prognosis	Ref
**BCAP31**	26.1%	Yes	(21)
**BORIS**	47.4%	Yes	(24)
**MAGE-A family**	27.2%-46.3%	Yes	(86–89)
**NY-ESO-1**	21%-30%	Yes	(91)
**SEMG1/2**	NA^*^	Yes	(49)
**NANOS3**	94.7%	Yes	(36)
**POTEE**	61%	Yes	(92)
**LY6K**	33.9%	Yes	(23)
**LAGE-1**	7.2%-32.1%	Yes	(89, 93)
**HORMAD1/2**	NA^*^	Yes	(94, 95)
**CT45**	13%-68.5%	Yes	(58)
**TSP50**	55%-67.7%	Yes	(55, 90)
**SPAG9**	52.5%	Yes	(96, 97)

NA^*^, Not available.

## Targeting Oncogenic CTAs for Lung Cancer Therapy

Due to their high antigenicity and tumour specificity, CTAs have been used as targets for lung cancer vaccines and in the development of therapeutics against cancer in recent decades.

### CTAs-Based Vaccines in Lung Cancer Prevention and Therapy

In view of the fact that lung cancer cells overexpress CTAs, researchers have explored whether a lung cancer cell lysate vaccine could induce immunity to CTAs. In one study, 21 thoracic malignant tumour patients were injected with 10 mg of a protein lysate vaccine of lung cancer cell line H1299. After 4 weeks, 57% of patients exhibited serologic responses to NY-ESO-1, one of the 21 patients developed antibodies to GAGE7, and four of the patients exhibited reactivity to XAGE and MAGE-C2, suggesting that immunity to CTAs can be easily induced by lung cancer cell lysate vaccine (98).

Vaccination with two epitope peptides derived from LY6K (also named URLC10) and cell division associated 1 (CDCA1) induced specific CTLs (Cytotoxic T lymphocytes) expressing various TCRs in vaccinated lung cancer patients. All LY6K-specific CTL clones showed Ca2+ influx, IFN-γ production and cytotoxicity when co-cultured with LY6K-pulsed tumour cells, indicating that CTA peptide-based vaccination induces antigen-specific CTLs (99).

Pedro et al. isolated HLA-A*02:01/CT37 peptide-specific TCR a and b chains from a lung cancer patient CD8+ T cell clone and constructed an innovative CD3z. These TCR chains, together with the engineered CD3z chain, were transduced into CD8+ T cells, where they induced CD8^+^ T cell cytotoxic activity and IFN-g secretion against peptide-pulsed autologous cells and HLA-A*02:01-positive and CT37-expressing lung cancer cell lines. This finding reveals a new strategy and method for developing innovative adoptive transfer immunotherapies against lung cancer (100).

### CTAs-Based Immune Cell Therapy

Sperm protein 17 (SP17) is expressed by lung cancer cells. Infection with the Ad-SP17 adenovirus induces higher levels of SP17 expression and significantly increases the frequency of CD80^+^, CD83^+^, CD86^+^ and HLA-DR^+^ dendritic cells (DC) that produce higher levels of IL-12. Co-culture of DC-Ad-SP17 with autologous lymphocytes induces high frequencies of IFNc^+^ and CD8^+^ CTLs, which have selective cytotoxicity against SP17^+^ lung cancer CRL-5922 cells in an HLA-I restrictive manner, suggesting that SP17-overexpression induces antigen-specific anti-tumour immunity against SP17^+^ NSCLC. Thus, SP17 may be a valuable target for the development of immunotherapy against SP17^+^ NSCLC (25, 26).

XAGE1 (also named GAGED2a) antibodies have been tested in lung cancer patients with or without the EGFR mutation. The overall survival of patients with XAGE1 antibodies in their serum was significantly prolonged in EGFR-mutated patients treated with EGFR-TKI and conventional chemotherapy, suggesting that endogenous XAGE1 antibodies have a synergistic effect with chemotherapeutics (101).

In another study, the spontaneous immune responses against XAGE-1b were observed in 10% of NSCLC patients and in 19% of stage IIIB/IV lung adenocarcinoma patients. In the antibody-positive patients, CD4 and CD8 T-cell responses were detected in 88% and 67% of patients, respectively. The CD4 T-cell clone recognizes DCs pulsed with the synthetic XAGE-1b protein or a lysate from XAGE-1b-transfected 293T cells. The CD8 T-cell clone shows cytotoxicity against tumours expressing XAGE-1b and the appropriate HLA class I allele, suggesting that XAGE-1b is an ideal target for a lung cancer vaccine and therapy (102, 103). Furthermore, antibody responses to recombinant L552S (an isoform of XAGE1) protein were observed in 7 of 17 lung pleural effusion fluids of lung cancer patients, strongly implying that the L552S protein is immunogenic. In short, these data suggest that XAGE-1b is a potential target for lung cancer therapy (104).

SUV39H2 histone lysine methyltransferase (SUV39H2), a novel cancer-testis antigen, is immunogenic and elicits cytotoxic CD8+ T-cell (CTL) responses against colon and lung cancer cells (105). For example, the primary blood dendritic cell lines (ihv-DCs), which are engineered to express MAGEA3 and high levels of 4-1BBL and MICA, induce simultaneous production of both HLA-A2-restricted, MAGEA3-specific CTLs and NK cells from HLA-A2+ donor peripheral blood mononuclear cells. These cytotoxic lymphocytes suppress lung metastasis of A549/A2.1 lung cancer cells in tumour-bearing mice (106). Therefore, oncogenic CTAs provide sensible targets for developing novel immunotherapy against lung cancer.

### CTA-Targeted Small Molecular Drugs for Lung Cancer Therapy

In normal somatic cells, most CTAs are silenced by DNA methylation (107). Silenced CTAs can be robustly reactivated by treatment with DNA methyltransferase inhibitors (DNMTi) (108, 109). A recent lung cancer therapy approach involves the induction of endogenously methylated CTAs by DNMTi in lung cancer to stimulate overexpression of CTAs with high antigenicity (23, 24).

5-Aza-2’-deoxycytidine (DAC) strongly induces overexpression of numerous CTAs. After treatment with DAC, the mRNA levels of meiosis specific with OB-fold (MEIOB), a novel CT antigen, is obviously increased; then, MEIOB antigen-specific T cells are produced in an HLA-restriction manner. Additionally, MEIOB peptide-specific helper T cells release IFN-γ in response to HLA-matched cancer cells, suggesting that the MEIOB peptide is an ideal small molecule for inducing immunity to lung cancer (110).

3-Deazaneplanocin A (DZNep) is a pharmacologic enhancer of zeste 2 polycomb repressive complex 2 subunit (KMT6) inhibitor, which suppresses histone methyltransferases. When lung cancer cells are treated with DZNep followed by DAC exposure, they are specifically recognized and lysed by allogeneic lymphocytes that express recombinant T cell receptors and recognize NY-ESO-1 and MAGE-A3, suggesting that a combination of DNA demethylating agents with histone demethylation drugs may provide a synergistic effect that induces CTA expression, which can be used as an adjunct to adoptive cancer immunotherapy (111).

### Oncogenic CTAs as Targets for Lung Cancer Chemotherapy

CTAs play important roles in cancer initiation and development. Accordingly, various CTAs have been targeted for lung cancer chemotherapy. CABYR is aberrantly overexpressed in lung cancer (112). The silencing of CABYR-a/b notably diminishes its downstream Akt pathway and decreases phospho-GSK-3b while upregulating protein levels of the tumour suppressors p53 and p27. Additionally, CABYR-a/b significantly increases the response to chemotherapeutic drugs and chemical drug-induced apoptosis *in vitro* and *in vivo*. Convincingly, overexpression of CABYR-a/b leads to constitutive activation of Akt signalling, partially restores the resistance to cisplatin and paclitaxel, and significantly decreases the amounts of cleaved PARP (53). These observations suggest that CABYR can be used as a target for lung cancer chemotherapy.

A peptide corresponding to the DEAD box domain (^266^AQTGTGKT^273^) of CAGE enhances the sensitivity of lung cancer cells to erlotinib and osimertinib by inhibiting the binding of CAGE to Beclin1 and regulating autophagic flux. The AQTGTGKT peptide elevates the expression levels of miR-143-3p and miR-373-5p, decreases autophagic flux and confers lung cancer cell sensitivity to anti-cancer drugs, implying that a combination of chemotherapeutics with CAGE-derived peptides may overcome drug resistance in non-small-cell lung cancers (27).

Cisplatin chemotherapy is one of the primary treatment modalities for NSCLC but can easily produce drug resistance in cancer patients. It has been reported that BORIS overexpression diminishes the DNA damage induced by cisplatin and is associated with a decreased overall survival rate in patients with NSCLC who have received cisplatin chemotherapy, suggesting that BORIS reduces cisplatin chemotherapy efficacy in NSCLC patients. Thus, the suppression of BORIS expression may be a new strategy for enhancing cisplatin chemotherapy sensitivity (113).

## Challenges, Opportunities and Perspectives

CTA research is providing new strategies and methods for lung cancer diagnosis and treatment; however, enormous challenges remain in CTA-based lung cancer treatment.

Because most CTA proteins are localized within the intracellular compartments of tumour cells, they cannot be directly recognized by specific antibodies or active immune cells. Cancer cells usually have defects in MHC class I protein (MHC-I)-mediated epitopes presenting on the cell surfaces; accordingly, only a small fraction of the intracellular CTAs is presented to the tumour cell surface, making CTA antigens available to either antibodies or immune cells and resulting in reduced efficacy of lung cancer immunotherapy (20).

**Figure 3:**
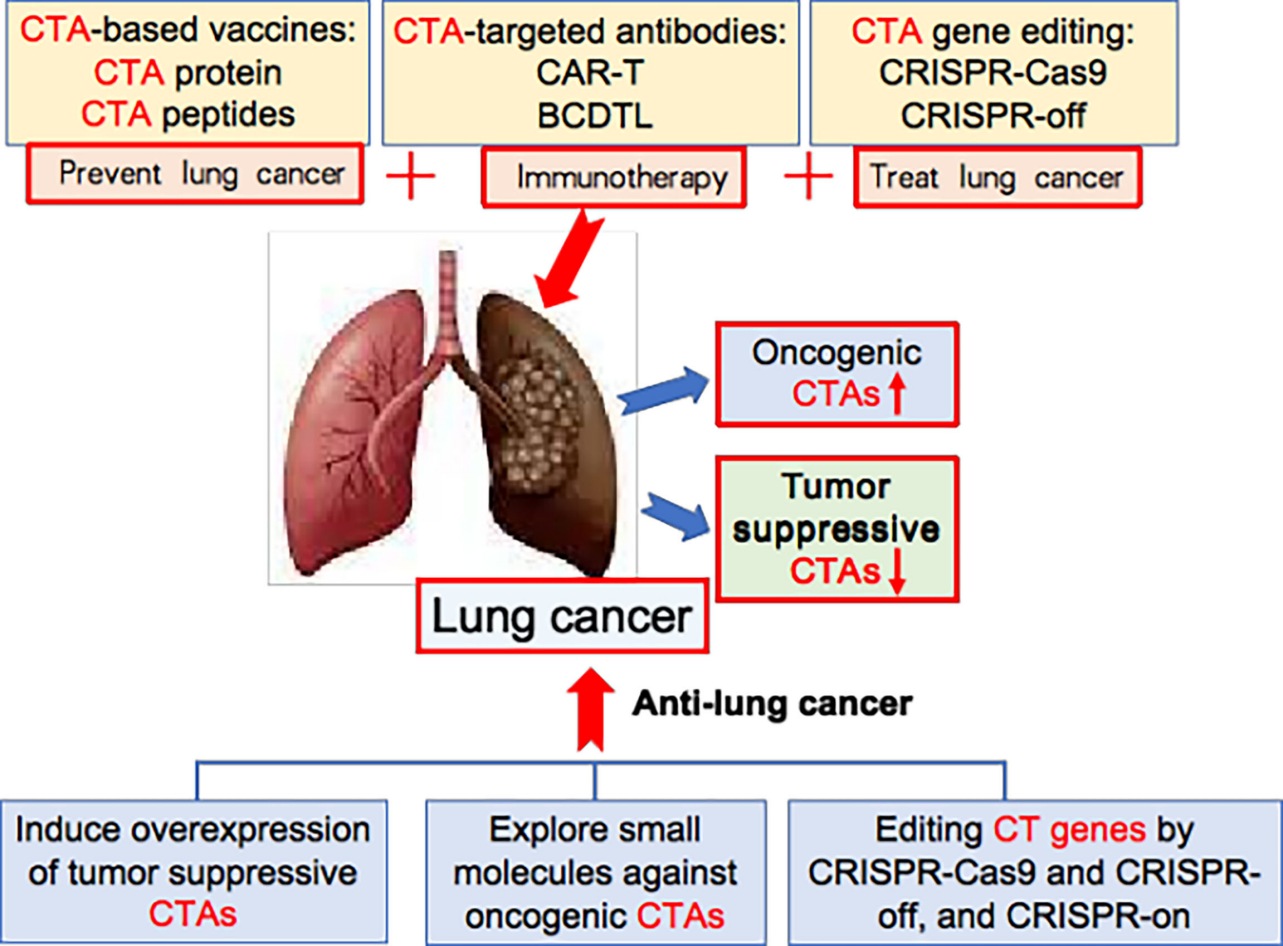
New strategy for CTA-based lung cancer therapy. The oncogenic CTAs are abnormally overexpressed in lung cancer, while tumour-suppressive CTAs are down-downregulated. CTA-based vaccines, CTA-targeted antibodies, and CTA gene editing are new ways for treating lung cancer. Additionally, inducing overexpression of tumour suppressive CTAs, exploring small molecules against oncogenic CTAs, and editing CT genes by CRISPR-Cas9 and CRISPR-off, and CRISPR-on provide new strategy for effective lung cancer therapy.

To increase the efficacy of lung cancer immunotherapy, we first need to identify and select CTAs that are located on cancer cell surfaces, and then raise specific antibodies against cell-surface CTAs for cancer therapy. In one study, human surface proteins were analysed by informatics tools. Some 22 potential cell-surface CTAs were identified, including ADAM30, C7orf45, OR11A1, SLC9A10 and SLCO6A1 (114). Secondly, we need to engineer cell-surface CTA-based Car-T to increase anti-lung cancer efficacy. Thirdly, we need new strategies that increase the efficiency of MHC-I-mediated CTA epitope presentation onto tumour cell surfaces, thus inducing strong immunity to lung cancer. Last but not least, in light of the fact that tumour-suppressive B cells in the tumour microenvironment are also involved in tumour immunity (20), we need to engineer lung cancer-suppressive B cell-dominated tertiary lymphoid structures in lung tumour tissues for effective cancer immunotherapy (**Figure 3**).

Although DNMT inhibitors, such as DAC, are strong CTA-inducer small molecules and are used in cancer treatment (115, 116), they concurrently induce the overexpression of a variety of CTAs. Notably, some of them are oncogenic, as stated before. Thus, the long-term net benefits and potential complications of DNMT inhibitor-mediated cancer therapy need to be evaluated and monitored.

In the last 10 years, gene editing (also called *genome editing*) has gained momentum in disease treatment. Gene editing is a new technology that can change an organism’s DNA and allows genetic material to be added, removed or altered at particular locations in the genome (117–121). Several approaches have been developed to edit the genome and epigenome, such as CRISPR-Cas9, CRISPR-off, CRISPR-on, and germinal choice technology (122–125). Various CTAs are abnormally overexpressed in tumour cells compared to normal cells, thereby giving us an opportunity to edit them in cancer cells precisely. Under this scenario, we propose several new strategies for CTA-targeted genetic and epigenetic editing for lung cancer therapy. Firstly, we may use CRISPR-Cas9 technology to knock out oncogenic CTAs; secondly, we may utilize CRISPR-off to methylate the gene-promoter region of oncogenic CTAs, thereby silencing these oncogenic CTAs; thirdly, we should explore tumour-suppressive CTAs, then use CRISPR-on technology to promote the overexpression of these tumour suppressors. (**Figure 3**) It is foreseeable that these strategies and cutting-edge technologies will lead to breakthroughs in CTA-targeted cancer therapy.

## Author Contributions

PY and QZ conceptualized and wrote the article. YQ conducted literature search and made Figures. MM participated in literature search and made tables in the article. All authors contributed to the article and approved the submitted version.

## Funding

This study was supported by grants from the National Natural Science Foundation of China (Grants No. 81572257, No. 81703595, and No. 81772535); State Key Laboratory of Radiation Medicine and Protection, School of Radiation Medicine and Protection; National Clinical Research Center for Hematologic Diseases (Grant No. 2020ZKMB04); a project funded by the Priority Academic Program Development of Jiangsu Higher Education Institutions (PAPD); the 2011 Collaborative Innovation Center of Hematology of Jiangsu Province; the Foundation of Medical School, Nantong University (Grants No. TDYX2021002).

## Conflict of Interest

The authors declare that the research was conducted in the absence of any commercial or financial relationships that could be construed as a potential conflict of interest.

## Publisher’s Note

All claims expressed in this article are solely those of the authors and do not necessarily represent those of their affiliated organizations, or those of the publisher, the editors and the reviewers. Any product that may be evaluated in this article, or claim that may be made by its manufacturer, is not guaranteed or endorsed by the publisher.
